# Screening for developmental delay at 18 months using the Infant Toddler Checklist: A validation study

**DOI:** 10.1371/journal.pone.0326751

**Published:** 2025-06-26

**Authors:** Cornelia M. Borkhoff, Haris Imsirovic, Imaan Bayoumi, Colin Macarthur, Kimberly M. Nurse, Teresa To, Mark E. Feldman, Eddy Lau, Braden Knight, Catherine S. Birken, Jonathon L. Maguire, Patricia C. Parkin

**Affiliations:** 1 Institute of Health Policy, Management and Evaluation, Dalla Lana School of Public Health, University of Toronto, Toronto, Ontario, Canada; 2 Child Health Evaluative Sciences, The Hospital for Sick Children Research Institute, Toronto, Ontario, Canada; 3 ICES, Toronto, Ontario, Canada; 4 Department of Family Medicine, Queen’s University, Kingston, Ontario, Canada; 5 Department of Pediatrics, Faculty of Medicine, University of Toronto, Toronto, Ontario, Canada; 6 MAP Centre for Urban Health Solutions, Li Ka Shing Knowledge Institute, Toronto, Ontario, Canada; American University of Beirut Medical Center, LEBANON

## Abstract

**Objective:**

The Infant Toddler Checklist (ITC) may be promising as a single tool at the 18-month visit to detect a range of developmental concerns. We examined the predictive validity of the ITC; and the association between positive ITC screening and health care utilization (HCU).

**Methods:**

Prospective cohort study of children at average-risk for developmental delay attending their 18-month visit in primary care in Toronto, Canada. Parents completed the ITC. HCU from the single-payer provincial health system was collected from health administrative databases ensuring complete follow-up. Physician billing code for a neurodevelopmental consultation was the primary outcome and criterion measure. Six other HCU types were assessed.

**Results:**

Of 1460 children with a mean age at screening of 18 months, 11% screened ITC positive. Mean age at follow-up was 8 years, 2.6% had a neurodevelopmental consultation. Screening test properties (with neurodevelopmental consultation as the criterion measure): 40% sensitivity (95% CI 24%, 57%), 90% specificity (95% CI 88%, 91%), 10% false positive rate (95% CI 9%, 12%). Using multivariable negative binomial regression, a positive ITC was associated with higher rates of 6 of 7 HCU types, including neurodevelopmental consultation (aRR 2.78, 95% CI 1.37, 5.67, p = 0.005).

**Conclusion:**

The ITC had high specificity and a low false positive rate, suggesting that most children with a negative ITC will not have a later neurodevelopmental consultation, and use of the tool may minimize unintended harms such as anxiety and resource use. The low sensitivity highlights the importance of ongoing developmental surveillance. Low sensitivity of other screening tools is discussed.

## Introduction

Professional organizations in many countries advocate for identification of developmental delays and disorders in early childhood through developmental surveillance and use of standardized developmental screening tools in primary care and public health settings [1–9]. Screening has been shown to be superior to surveillance for identification, referral, and eligibility for intervention services [10]. The American Academy of Pediatrics (AAP) recommends that all children with positive screening should receive a referral for a medical and developmental evaluation [1]. While developmental surveillance is a long-standing clinical practice, there is international variation in the implementation of developmental screening with regard to age of screening and type of screening tool [1–9].

Developmental screening may occur at multiple ages or once at a specific age. For example, the American Academy of Pediatrics recommends screening at multiple ages (9, 18, 24, 30 months) [1,2]; the Canadian Paediatric Society recommends screening only once at 18 months [3]; and the U.K. Healthy Child Programme recommends screening at 2 years [7,8]. Despite these differences, screening at 18–24 months is recommended, highlighting the importance of this age at which delays in communication and language development are often evident [1].

Types of screening tools include general/broadband tools and domain/disorder-specific tools (including autism spectrum disorder [ASD]-specific tools). At the 18-month visit, the AAP recommends a general tool plus an ASD-specific tool [1,2]. A survey of American pediatricians found that commonly used screening tools are the Ages and Stages Questionnaire (ASQ) as a general tool and the Modified Checklist for Autism in Toddlers (M-CHAT) as an ASD-specific tool [11]. However, there is some evidence that these tools have low sensitivity and positive predictive value in younger (18–24 months) average-risk children [12–15]; and a recent study showed that a positive screen on the ASQ communication domain was a stronger predictor of referral than a positive M-CHAT screen [16]. This raises the question of the need for two types of screening tools to identify children needing referral. Administration of two screening tools may be burdensome and is not recommended in other countries such as Canada and the U.K.

In this context, the Infant Toddler Checklist (ITC) may be promising as a single screening tool at the 18-month visit. The ITC developers have reported its ability to detect a range of developmental concerns, including language delay, global developmental delay, and ASD [17–20]. The ITC is freely available, takes 5 minutes for parents to complete and 2 minutes for scoring [21]. The feasibility of administering the ITC at 18 months has been assessed by 203 primary care pediatricians in over 40,000 children in California [21,22]; and in over 2,600 children in publicly-funded Swedish child health services [23,24]. However, the validity of the ITC in routine primary care has not been fully evaluated.

The validity of developmental screening tools may be assessed with measurement of the tool and criterion at the same time (concurrent validity) or measurement of the criterion at some time after the tool (predictive validity) [25]. Concurrent validity studies often use a diagnostic assessment as the criterion measure [12,13,26–29]. Predictive validity studies examine future outcomes that are meaningful to children, families, practitioners and the health care system as the criterion measure [25,30,31]. Tool accuracy is influenced by child age at screening, with lower sensitivity at younger compared with older age; risk for developmental delay, with lower sensitivity in average-risk compared with high-risk children; and diagnostic assumptions made for children lost to follow-up, with higher sensitivity when lower prevalence of diagnosis assumed [26,30,32]. For application in our primary care and public health settings, we aimed to assess the predictive validity of the ITC in average-risk children screened at 18 months of age with complete follow-up at a later age.

Developmental screening tools are not intended to be used as a diagnostic assessment [33]. Rather, these tools are intended to identify children who should receive close monitoring (i.e., a follow-up visit) or referral (i.e., for a diagnostic assessment), as recommended by the AAP [1]. It has also been emphasized that screening tools must not only be accurate but also improve care by influencing clinician and parent decision-making [32]. Therefore, health care utilization for consultation and monitoring are meaningful outcomes for assessing the predictive validity of a screening tool and its association with clinical care.

We hypothesized that children with developmental concerns identified in early childhood would be more likely to receive a neurodevelopmental consultation; and that children requiring close monitoring and/or referral would have greater health care utilization including unscheduled primary care visits and non-primary care and specialist visits.

Our primary objective was to examine the predictive validity of the ITC in average-risk children at the 18-month primary care visit, using neurodevelopmental consultation as the criterion measure. The secondary objective was to examine the association between positive screening using the ITC at the 18-month visit with later health care utilization (HCU).

## Methods

### Design

We used a prospective cohort study design. Developmental screening results from parent-completed ITC were collected at the child’s 18-month visit in primary care. Later neurodevelopmental consultation and other HCU were collected from health administrative databases. We followed the Standards for Reporting of Diagnostic Accuracy (STARD) guideline [34]. The study period was October 1, 2011 to March 31, 2021.

### Setting and usual care

The study was embedded in real-world primary care practices participating in the *TARGet Kids!* practice-based research network (www.targetkids.ca) in the province of Ontario, Canada, where primary care is delivered through the universal health care system by both pediatricians and family physicians. The Canadian Paediatric Society recommends 11 health supervision visits from birth to 5 years [35] and use of a developmental screening tool once at the 18-month visit [3]. Beginning in 2009, as part of a physician incentive program, the provincial government provided the Nipissing District Developmental Screen (NDDS) free of charge which has been widely used as the developmental screening tool at the 18-month visit [3,36,37]. Despite its widespread use, the NDDS has been reported to have poor performance characteristics and in 2023 the province announced they would not be renewing the province-wide license and providing the NDDS free-of-charge [36,38]. However, as our study period preceded 2023, and in keeping with usual practice, parents completed the NDDS which was used by the child’s physician for clinical care purposes.

Referral is required for neurodevelopmental assessments which are completed by developmental and consultant pediatricians. During this study, usual care (including developmental surveillance, screening using the NDDS, and later health care utilization such as monitoring and referral) was conducted as per the child’s primary care physician, with no involvement by the research team.

### Data sources

The *TARGet Kids!* database was the source for participant characteristics and ITC results which were collected prospectively. At the 18-month visit, parents completed a standardized data collection form and the ITC. The *TARGet Kids!* cohort profile has been published and registered at ClinicalTrials.gov (NCT01869530) [39].

Population-based health administrative databases held at ICES (Ontario, www.ices.on.ca) were the source for HCU data. ICES is an independent, not-for-profit research institute which holds health data that are routinely collected for the publicly-funded, single-payer healthcare system in the province of Ontario, Canada. Use of these data is authorized under Ontario’s Personal Health Information Protection Act. Several ICES databases were used as the source for HCU data: The Ontario Health Insurance Plan (OHIP) physician claims database includes physician fee-for-service billings; the Canadian Institute for Health Information Discharge Abstract Database (DAD) includes acute-care hospitalizations; the National Ambulatory Care Reporting System (NACRS) includes emergency department (ED) visits. Data were available from January 1, 2010 to March 31, 2022.

Data from children in *TARGet Kids!* were deterministically linked at the individual level using each child’s encrypted OHIP number to ICES databases. Using data from this single-payer system ensured complete follow-up of all participants.

### Participants

Children were eligible if they were 16–23 months of age attending a scheduled 18-month health supervision visit and had a parent-completed ITC between October 1, 2011 and July 31, 2019.

To ensure a study population at average-risk for developmental delay, we excluded children with health conditions affecting growth and development, established diagnosis of developmental delay, gestational age < 35 weeks, and birthweight <2.5 kg; as well as unscheduled visit, parents unable to communicate in English, no valid OHIP, not continuously eligible for OHIP between birthdate and index screening or between index screening and 1 year post screening, index date after March 31, 2021, or did not have at least 1 year of follow-up.

### Infant Toddler Checklist

Wetherby and Prizant developed the ITC for children ages 6–24 months (see the checklist and scoring available online) [17] and evaluated the concurrent and predictive validity in non-primary care settings [18,19]. In children 12–24 months, the developers reported a sensitivity of 83%−89% and specificity of 70%−77% for a communication disorder, and a sensitivity of 93% for ASD [19,20].

The ITC includes 24 items which yield three composite scores (social, speech, symbolic) and their sum produces a total score [17–20]. The 10^th^ percentile cut-off for each month of age indicates concern/no concern for each of the four scores (three composite scores and total score).

For this study, parents completed the ITC once at the 18-month visit. The ITC was completed for research purposes; physicians and parents were blind to the results. We examined three components of the ITC using definitions from the developers’ recommendations, which were included as predictors in our analytical models. First, a positive ITC was defined as concern for speech delay (defined as concern on the speech composite), or concern for other communication delay (defined as concern on the social composite, symbolic composite, or total score), or concern for both. Then, we separately examined: concern for speech delay (recommendation: re-screen in 3 months to determine if referral is advisable) and concern for other communication delay (recommendation: refer).

### Neurodevelopmental consultation

The physician billing code for a neurodevelopmental consultation (at least once at any time in the follow-up period) was the primary outcome and criterion measure. The Ontario Ministry of Health Schedule of Benefits defines a neurodevelopmental consultation (billing code A667, fee CAD$401.30) as: “a consultation in which the physician provides all the elements of a consultation (A265) for an infant, child or adolescent with complex neurodevelopmental conditions (e.g., autism, global developmental disorders, etc.) and spends a minimum of 90 minutes of direct contact with the patient and caregiver. The service is limited to a maximum of one per patient, per physician, per 12-month period” [40]. We have recently published an analysis of the validity of the NDDS using this same criterion [38].

### Other health care utilization

For our secondary objective to assess other HCU at any time in the follow-up period, which may represent monitoring and/or referral, the following were collected: special pediatric consultation/assessment (at least once), scheduled primary care visit billings (cumulative number of billings), unscheduled primary care or minor visit billings (cumulative number of billings), other non-primary care visit billings (cumulative number of billings), hospitalizations (0, 1, 2+), ED visits (0, 1, 2+). The category special pediatric consultation/assessment includes two physician billing codes for extended pediatric consultation (75–90 minutes) and three specialized codes for developmental and/or behavioral care. Billing codes were grouped by three experienced primary care physicians by consensus. See S1 Table for physician billing codes.

### Statistical analysis

Descriptive statistics were used to characterize all participants, as well as for those children who screened positive and who screened negative. We also described health care utilization at baseline from birth until index screening using the ICES data prior to index date (i.e., the look-back window).

To examine the predictive validity of the ITC, we calculated the screening test properties (sensitivity, specificity, false positive rate, positive predictive value [PPV], negative predictive value [NPV]), with 95% confidence intervals (CIs), for each of the three components of the ITC at screening. We used the neurodevelopmental consultation physician billing code as the criterion measure.

We also examined the association between ITC (predictor) with HCU (outcome) using multivariable negative binomial regression analyses. The logarithm of observation time was used as an offset to account for variation in the window of observation. For our binary outcomes (e.g., neurodevelopmental consultation), the offset was the time to first event of outcome or end of follow-up, for those without the outcome. For count outcomes (e.g., scheduled primary care visits), the offset was the entire time a child spent in the study. We used separate regression models to estimate the rate ratio (RR) and 95% CIs for each of the 7 HCU outcomes, which were measured after the index date when the ITC was administered. Models were adjusted for child age, child sex, number of siblings, maternal ethnicity, self-reported annual family income (CAD$), family immigration status, and family history of developmental concern (ASD, attention deficit disorder, learning disability) regardless of statistical significance [41]. All covariates were measured at index screening. Child age and child sex had complete data. All other covariates had < 12% missing data, except for family history of developmental concern, which had 22% missing data; multiple imputation by chained equation (MICE) was used to impute missing covariate data [42]. Statistical significance was defined as p < 0.05; all statistical tests were two-sided. Statistical analysis was conducted using SAS V.9.4 statistical software (SAS Institute).

### Ethics

Ethics approval was obtained from the Hospital for Sick Children and Unity Health Toronto Research Ethics Boards, Toronto, Canada. Parent and/or legal guardian informed written consent was obtained. For clinical care, physicians followed usual care as described in ‘Setting and usual care’.

## Results

### Participants

**Fig 1:**
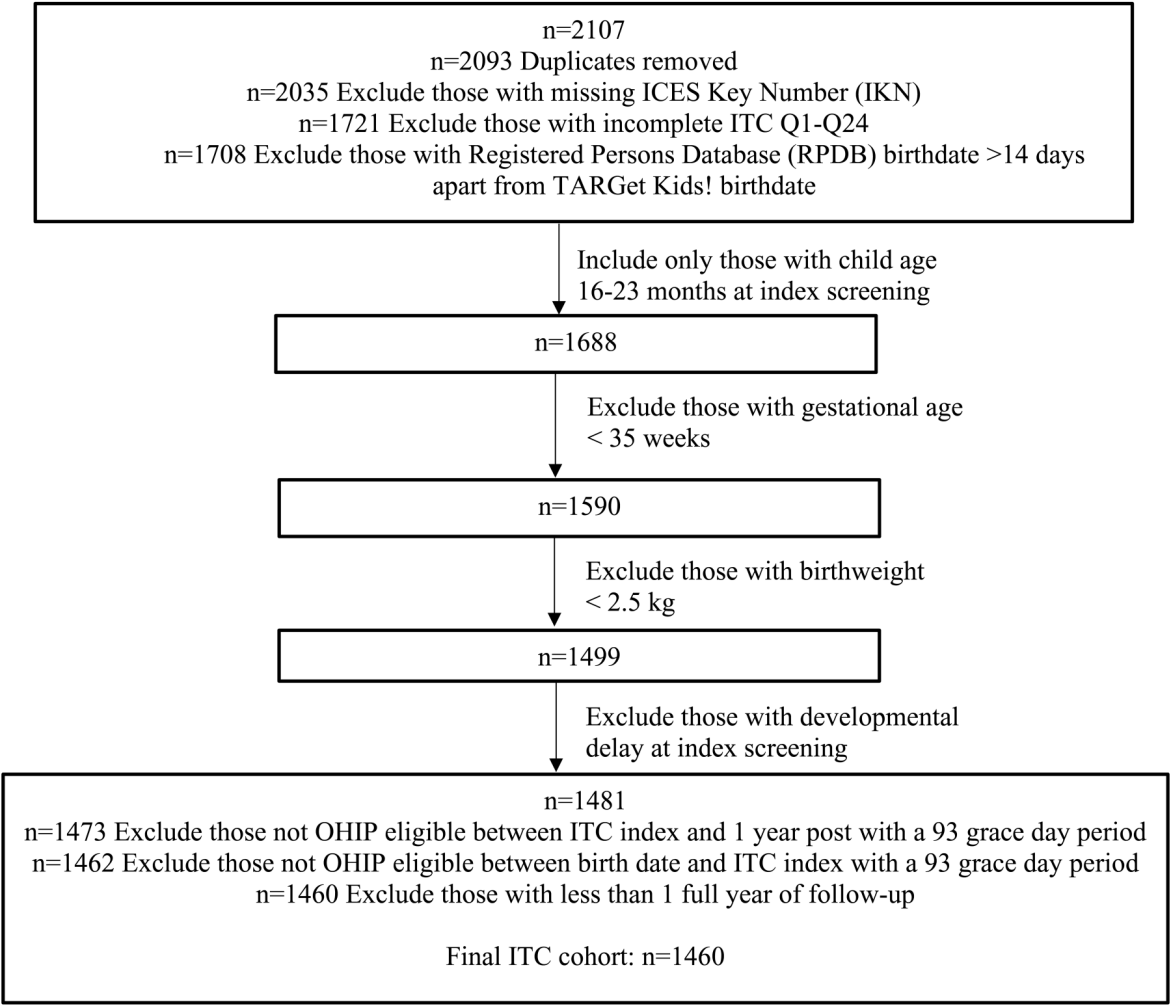
Study participant flow chart.

Fig 1 shows the participant flow for the final cohort (n = 1,460, mean age 18.2 months). Table 1 shows the participant characteristics at index screening, and HCU from birth until index screening. Of 1,460 children screened, 160 (11.0%) screened positive. Table 2 shows HCU from index screening to follow-up. The mean (SD) time to follow-up was 6.5 (2.2) years, 38 (2.6%) had a neurodevelopmental consultation, and 165 (11.3%) had a special pediatric consultation/assessment.

**Table 1:** Participant characteristics at index screening with the Infant Toddler Checklist (n = 1,460) and health care utilization from birth until index screening.

Characteristics	Infant Toddler Checklist			
	All Participants		Positive^a^	Negative
**N (%)**	N	1460	160 (11.0)	1300 (89.0)
**Child and family characteristics**				
Age, months, mean (SD)	1460	18.2 (1.0)	18.3 (1.1)	18.2 (1.0)
Female sex, n (%)	1460	663 (45.4)	47 (29.4)	616 (47.4)
Birthweight, kg, mean (SD)	1456	3.4 (0.5)	3.4 (0.4)	3.4 (0.5)
zBMI, mean (SD)	1430	0.2 (1.1)	0.2 (1.2)	0.2 (1.1)
Siblings, n (%)	1418			
0		725 (51.1)	65 (41.4)	660 (52.3)
1		536 (37.8)	63 (40.1)	473 (37.5)
2+		157 (11.1)	29 (18.5)	128 (10.2)
Maternal age at birth, years, mean (SD)	1368	33.9 (4.3)	33.0 (5.5)	34.0 (4.1)
Maternal ethnicity, n (%)	1283			
European		799 (62.3)	63 (43.8)	736 (64.6)
Non-European		484 (37.7)	81 (56.3)	403 (35.4)
Maternal education, n (%)	1433			
High school or less		110 (7.7)	23 (14.7)	87 (6.8)
College/University		1323 (92.3)	133 (85.3)	1190 (93.2)
Self-reported family income (CAD $), n (%)	1413			
Less than $40,000		126 (8.9)	38 (24.7)	88 (7.0)
$40,000 to $79,999		179 (12.7)	28 (18.2)	151 (12.0)
$80,000 to $149,999		485 (34.3)	41 (26.6)	444 (35.3)
$150,000 or more		623 (44.1)	47 (30.5)	576 (45.8)
Family immigration status, n (%)	1402			
Canadian-born		766 (54.6)	62 (40.3)	704 (56.4)
Immigrant, industrialized		192 (13.7)	13 (8.4)	179 (14.3)
Immigrant, non-industrialized		444 (31.7)	79 (51.3)	365 (29.2)
Single parent household, n (%)	1428	57 (4.0)	11 (7.0)	46 (3.6)
Family history of developmental concern, n (%)	1132	96 (8.5)	14 (10.9)	82 (8.2)
**Health Care Utilization, from birth to index screening**				
Special pediatric consultation/assessment, n (%)	1460	40 (2.7)	1-5^*^	35-39^*^
Scheduled primary care visit billings, n (%)	1460			
≤ 10		37 (2.5)	1-5^*^	32-36^*^
11-20		735 (50.3)	71 (44.4)	664 (51.1)
21-30		637 (43.6)	73 (45.6)	564 (43.4)
31+		51 (3.5)	11-15^*^	36-40^*^
Unscheduled primary care or minor visit billings, n (%)	1460			
0		853 (58.4)	78 (48.6)	775 (59.6)
1		369 (25.3)	49 (30.6)	320 (24.6)
2		135 (9.2)	15 (9.4)	120 (9.2)
3+		103 (7.1)	18 (11.3)	85 (6.5)
Other non-primary care visit billings, n (%)	1460			
≤ 10		625 (42.8)	61 (38.1)	564 (43.4)
11-20		457 (31.3)	44 (27.5)	413 (31.8)
21-30		209 (14.3)	27 (16.9)	182 (14.0)
31-40		86 (5.9)	12 (7.5)	74 (5.7)
41+		83 (5.7)	16 (10.0)	67 (5.2)
Hospitalizations, n (%)	1460			
0		66 (4.5)	1-5^*^	61-65^*^
1		1203 (82.4)	131 (81.9)	1072 (82.5)
2+		191 (13.1)	24-28^*^	163-167^*^
ED visits, n (%)	1460			
0		737 (50.5)	76 (47.5)	661 (50.8)
1		394 (27.0)	43 (26.9)	351 (27.0)
2+		329 (22.5)	41 (25.6)	288 (22.2)

Data are presented as n (%) or mean (SD). SD = standard deviation

* Data are suppressed due to small cell size.

^a^We defined a positive ITC screen as concern for speech delay and/or concern for other communication delay

**Table 2:** Health care utilization from index screening at 18 months to follow up for children screened using the Infant Toddler Checklist (n = 1,460).

Outcome	Infant Toddler Checklist		
	All Participants	Positive^a^	Negative
**N**	1460	160 (11.0)	1300 (89.0)
Follow-up, years, mean (SD)	6.5 (2.2)	6.5 (2.0)	6.5 (2.2)
**Primary Outcome**			
Neurodevelopmental consultation, n (%)	38 (2.6)	15 (9.4)	23 (1.8)
**Secondary Outcomes**			
Special pediatric consultation/assessment, n (%)	165 (11.3)	35 (21.9)	130 (10.0)
Scheduled primary care visit billings, n (%)			
≤ 10	604 (41.4)	61 (38.1)	543 (41.8)
11-20	631 (43.2)	62 (38.8)	569 (43.8)
21-30	162 (11.1)	22 (13.8)	140 (10.8)
31+	63 (4.3)	15 (9.4)	48 (3.7)
Unscheduled primary care or minor visit billings, n (%)			
0	634 (43.4)	57 (35.6)	577 (44.4)
1	374 (25.6)	37 (23.1)	337 (25.9)
2	207 (14.2)	21 (13.1)	186 (14.3)
3+	245 (16.8)	45 (28.1)	200 (15.4)
Other non-primary care visit billings, n (%)			
≤ 10	279 (19.1)	26 (16.3)	253 (19.5)
11-20	311 (21.3)	22 (13.8)	289 (22.2)
21-30	242 (16.6)	33 (20.6)	209 (16.1)
31-40	165 (11.3)	23 (14.4)	142 (10.9)
41+	463 (31.7)	56 (35.0)	407 (31.1)
Hospitalizations, n (%)			
0	1192 (81.6)	129 (80.6)	1063 (81.8)
1	206 (14.1)	21 (13.1)	185 (14.2)
2+	62 (4.2)	10 (6.3)	52 (4.0)
ED visits, n (%)			
0	428 (29.3)	45 (28.1)	383 (29.5)
1	355 (24.3)	39 (24.4)	316 (24.3)
2+	677 (46.4)	76 (47.5)	601 (46.2)

Data are presented as n (%) or mean (SD). SD = standard deviation

^a^We defined a positive ITC screen as concern for speech delay and/or concern for other communication delay

### Predictive validity

The screening test properties with neurodevelopmental consultation as the criterion measure are shown in Table 3. A positive ITC screen had a sensitivity of 39.5% (95% CI 24.0%, 56.6%), specificity of 89.8% (95% CI 88.1%, 91.3%), false positive rate of 10.2% (95% CI 8.6%, 12.0%), PPV of 9.4% (95% CI 5.3%, 15.0%), and NPV of 98.2% (97.4%, 98.9%). Low sensitivity and high specificity were also found for concern for speech delay and concern for other communication delay.

**Table 3:** Screening test properties of the 18-month Infant Toddler Checklist (n = 1,460) at the 18-month visit compared with later neurodevelopmental consultation at mean follow-up of 6.5 years.

Infant Toddler Checklist (n = 1460)									
Screening at 18 months	Neurodevelopmental Consultation, yes n = 38		Neurodevelopmental Consultation, no n = 1422		Sensitivity	Specificity	False Positive Rate	PPV	NPV
	True Positive (TP)	False Negative (FN)	False Positive (FP)	True Negative (TN)					
Concern for speech delay and/or concern for other communication delay	15	23	145	1277	39.5 (24.0, 56.6)	89.8 (88.1, 91.3)	10.2 (8.6, 12.0)	9.4 (5.3, 15.0)	98.2 (97.4, 98.9)
Concern for speech delay	9	29	84	1338	23.7 (11.4, 40.2)	94.1 (92.7, 95.3)	5.9 (4.7, 7.3)	9.7 (4.5, 17.6)	97.9 (97.0, 98.6)
Concern for other communication delay	12	26	93	1329	31.6 (17.5, 48.7)	93.5 (92.1, 94.7)	6.5 (5.3, 8.0)	11.4 (6.1, 19.1)	98.1 (97.2, 98.7)

All values for screening test properties presented as percentage (95% CI).

Abbreviations: CI, confidence interval; PPV, positive predictive value; NPV, negative predictive value.

The adjusted rate ratios (aRR) are shown in Table 4. A positive ITC screen was associated with higher rates of neurodevelopmental consultation (aRR 2.78, 95% CI 1.37, 5.67, p = 0.005), special pediatric consultation/assessment (aRR 1.75, 95% CI 1.17, 2.61, p = 0.007), scheduled primary care visit billings (aRR 1.11, 95% CI 1.02, 1.21, p = 0.02), unscheduled primary care or minor visit billings (aRR 1.44, 95% CI 1.14, 1.81, p = 0.002), other non-primary care visit billings (aRR 1.15, 95% CI 1.00, 1.32, p = 0.05), and hospitalizations (aRR 1.48, 95% CI 1.01, 2.19, p = 0.046) compared with a negative ITC screen.

**Table 4:** Association between positive developmental screening at 18 months using the Infant Toddler Checklist (n = 1,460) and later health care utilization.

Outcome	Infant Toddler Checklist			
	Unadjusted analysis		Adjusted analysis^*^	
	RR (95% CI)	P value	aRR (95% CI)	P value
**Primary outcome**				
Neurodevelopmental consultation	5.31 (2.77, 10.17)	<0.0001	2.78 (1.37, 5.67)	0.005
**Secondary outcomes**				
Special pediatric consultation/assessment	2.19 (1.51, 3.18)	<0.0001	1.75 (1.17, 2.61)	0.007
Scheduled primary care visit billings	1.13 (1.04, 1.22)	0.006	1.11 (1.02, 1.21)	0.02
Unscheduled primary care or minor visit billings	1.81 (1.46, 2.25)	<0.0001	1.44 (1.14, 1.81)	0.002
Other non-primary care visit billings	1.21 (1.06, 1.38)	0.005	1.15 (1.00, 1.32)	0.05
Hospitalizations	1.65 (1.15, 2.37)	0.006	1.48 (1.01, 2.19)	0.046
ED visits	1.22 (1.02, 1.46)	0.03	1.11 (0.92, 1.33)	0.29

Abbreviations: RR = rate ratio; aRR = adjusted rate ratio; CI = confidence interval

* Models were adjusted for child age at index screening, child sex, number of siblings, maternal ethnicity, self-reported family income (CAD$), family immigration status, family history of developmental concern regardless of statistical significance. All covariates were measured at baseline. Child age and child sex had complete data. All other covariates had < 12% missing data, except for family history of developmental concern, which had 22% missing data; multiple imputation by chained equation (MICE) was used for missing data.

## Discussion

In this study of 1,460 children receiving developmental screening at 18 months, 11% had a positive ITC (similar to rates in California [14%] and Sweden [13%]) [22,24], and 2.6% received a neurodevelopmental consultation at an average age of 8 years. We examined both tool accuracy and influence on clinical decision-making [32]. A positive ITC had high specificity (90%) and low false positive rate (10%) but low sensitivity (40%). This suggests that most children with a negative ITC will not have a later neurodevelopmental consultation (specificity), but the tool cannot accurately identify those who will have a later neurodevelopmental consultation (sensitivity) resulting in many false negatives. The low false positive rate may minimize unintended harms such as anxiety and resource use [43]. A positive ITC at 18 months was associated with higher rates of six of seven types of health care utilization measured at approximately 8 years. This suggests a strong association between the ITC and clinical decision-making with respect to children’s need for health care services, given that physicians and parents were blind to the results of the ITC.

Pierce and colleagues have reported on outcomes and feasibility of implementing the ITC in a network of 203 primary care pediatricians in San Diego County and established a system for screening, evaluation, and referral for neurodevelopmental risk [21,22]. The investigators selected the ITC as the screening tool due to its ability to identify children with a range of developmental concerns, including young children with ASD who do not exhibit full symptoms and who may be missed using ASD-specific screening tools [22]. Over 40,000 children were screened at 12, 18, and/or 24 months (median age of 18 months), 14% had a positive ITC screen, and 2% were referred and completed at least one diagnostic evaluation (median age of 20 months) [22]. Following evaluation, children received a range of diagnoses including language delay, global developmental delay, and ASD or ASD features. Of pediatricians completing a satisfaction survey, 96% provided a positive evaluation [21]. The authors reported a positive predictive value of 75%, but noted that their studies were not designed to assess sensitivity and specificity which could not be calculated due to incomplete follow-up of all screen positive and screen negative children [21]. The authors have also reported that the mean age of screening and evaluation was similar among children of diverse ethnic or racial backgrounds, suggesting their system may ensure equitable access to care for all children [44]. This system of screening using the ITC followed by diagnostic evaluation has been successfully used for other clinical and genetic studies of ASD [45,46].

Fäldt and colleagues have reported on the implementation of the ITC at the 18-month visit in publicly-funded Swedish child health services using the RE-AIM framework (reach, effectiveness, adoption, implementation, maintenance) [23,24]. Of 2,633 children screened, 13% had a positive ITC and the referral rate to a speech and language pathologist increased from 0.4% pre-implementation to 6.9% post-implementation [24]. However, calculation of concurrent validity of the ITC was limited as not all children with a positive screen and only a random sample of children with a negative screen were referred and assessed with the criterion measure [23].

We previously examined the predictive validity of the ITC using two different criterion measures: parent-reported developmental diagnosis at 3–5 years (n = 488), and teacher-reported school readiness at 4–6 years (n = 293) [47,48]. The previous analyses showed comparable sensitivity, specificity and false positive rates despite different criterion measures and prevalence rates (prevalence: 5.3% for parent-reported developmental diagnosis, 18.4% for school readiness vulnerability). Our current analysis provides a larger sample size (n = 1460) and a health systems related criterion measure (physician billing for a neurodevelopmental consultation, prevalence 2.6%). In addition to evaluating predictive validity of the ITC using a variety of criterion measures, we have examined factors associated with a positive ITC, and found an association with low family income, child male sex, and low birthweight, providing additional evidence of ‘known-groups’ validity of the ITC [49].

Strengths of the ITC include its focus on communication, which is highly relevant to developmental milestones at the 18-month visit, the easy-to-complete 1-page format, accessibility free-of-charge, and ability to detect a range of developmental concerns (language delay, global developmental delay, and ASD). The ITC does not screen specifically for motor delays and disorders. To address this limitation, a milestone approach to developmental surveillance, including physical examination and history, may lead to identification of motor delays without the use of a screening tool [1,50]. A recent study examining the predictors of early intervention referral after a positive developmental screen in primary care, reported no association with the scores on the gross motor domain of the ASQ [16].

Other general and ASD-specific screening tools are widely used. Review of the accuracy of these tools reveals low sensitivity and high specificity when used in young (18–24 months), average-risk children, especially when predictive validity is assessed (described as the ‘thorny nature’ of predictive validity studies of developmental screening), and complete follow-up is ensured [25,26,32].

The Ages and Stages Questionnaires (ASQ), now in its third edition (ASQ-3), is a general development tool for children 1–66 months, and screens 5 domains: communication, gross motor, fine motor, problem solving and personal-social [51]. Screening test properties have been evaluated in several recent systematic reviews [27,28,30,31]. However, reported summary measures of sensitivity and specificity may be misleading, as included studies represent a wide age range (0–60 months), mixed populations (average- and high-risk), and various criterion measures. Sheldrick et al examined concurrent validity of the ASQ-3, compared with the Bayley Scales of Infant and Toddler Development (BSID-III), in children 0–42 months recruited from primary care offices and reported a sensitivity of 35% and specificity of 89% [12]. Veldhuizen et al examined the concurrent validity of the ASQ, compared with the BSID-III, in a community sample of children 1–36 months (n = 587) and reported a sensitivity of 41% and specificity of 82% [13]. Solgi et al reported that only the communication domain was associated with referral to early intervention following screening with the ASQ-3 at 18–24 months in primary care [16].

The Modified Checklist for Autism in Toddlers, Revised with Follow-Up (M-CHAT-R/F) is an ASD-specific tool for children ages 16–30 months and a risk score determines if a follow-up structured questionnaire is required [52]. Screening test properties have been evaluated in two recently published systematic reviews [26,29]. Aishworiya et al [26] included 15 studies; however, summary measures of sensitivity and specificity were based on limited follow-up and evaluation of screen-negative children [53]. Using results from a published large-scale screening study [52], Sheldrick et al developed simulation models with six scenarios, each using different assumptions regarding loss to follow-up, and reported a sensitivity ranging from 40% to 94% across scenarios [32]. Wieckowski et al [29] included 50 studies; however, sensitivity varied widely according to child age at screening (14–86 months), population (average- or high-risk), and design (concurrent or predictive). In two recent predictive validity studies of average-risk children 18–24 months in primary care, sensitivity was 33–39% and specificity was 94–98% [14,15].

Strengths of our study include a large sample size, recruitment of average-risk children from primary care practices during scheduled health supervision visits, recruitment of families with diverse characteristics (included in the adjusted analyses), blinding of parents and physicians to the ITC results, use of standardized physician billing codes, and predictive validity design. Whereas many studies are limited by the challenges of completing follow-up and evaluation of all screened children [26,32,33,53], we were able to ensure complete follow-up of both screen positive and screen negative children by linking individual parent-reported data and population-based health administrative databases.

Limitations include the use of physician billing codes for neurodevelopmental consultation rather than diagnosis codes for developmental disorders (which have not been validated); however, the aim of screening is to identify children who should receive monitoring or referral, and other predictive validity studies have used outcomes other than diagnosis, such as educational outcomes and referral to early intervention [16,30,31]. While billing code algorithms have not been validated, these codes are likely to be more accurate as they represent a remunerative event rather than a subjective clinical impression. Our study was also limited by exclusion of parents unable to communicate in English. Given our aim to assess ITC validity in average-risk 18-month-old children to predict physician health care, we were unable to report on ITC validity in high-risk children, older children, or prediction of non-physician health care (such as speech-language therapy).

## Conclusion

There are several implications for practice and policy. First, screening at 18 months using the ITC has high specificity and a low false positive rate. Second, the ITC has low sensitivity, consistent with current literature showing low sensitivity of other commonly used screening tools when examined prospectively in young, average-risk children in real-world primary care settings; this highlights the importance of ongoing developmental surveillance beyond the 18-month visit. Third, screening with a single tool which may identify children with a range of developmental concerns, rather than administering two tools, may be an efficient approach warranting further study.

## Supporting information

S1 Table OHIP fee codes used to define study outcomes. (DOCX)
