# Supplementary material for: Screening for developmental delay at 18 months using the Infant Toddler Checklist: A validation study
Source: PLoS One. 2025 Jun 26;20(6):e0326751. doi: 10.1371/journal.pone.0326751 (PMC12200864; doi:10.1371/journal.pone.0326751)
Supplement: S1 Table — (DOCX) [file pone.0326751.s001.docx]

**S1 Table**. OHIP fee codes used to define study outcomes

|  |  |
| --- | --- |
| ***Primary Outcome*** |  |
| Neurodevelopmental Consultation | A667 = Neurodevelopmental Consultation - $401.30 (90 minutes) |
| ***Secondary Outcomes*** |  |
| Special pediatric consultation/assessment | A260 = Special Pediatric Consultation - $310.45 (75 minutes)  A662 = Extended Special Pediatric Consultation - $401.30 (90 minutes)  K119 = Pediatric Developmental Assessment Incentive - $115.10 (Ongoing management of a child <6 years at developmental risk using a surveillance approach)  Note: No billings for either of these 2 codes considered Specialist-Pediatric:  K122 = Individual developmental and/or behavioural care – $89.70  K123 = Family developmental and/or behavioural care – $101.75 |
| Scheduled primary care visit billings | A002 = FAMILY PRACTICE & PRACTICE IN GENERAL - ENHANCED 18 MONTH WELL BABY VISIT  A007 = INTERMED.ASSESS/WELL BABY CARE-F.P./G.P./PAED.  A262 = LEVEL 2 - PAEDIATRIC INTERMEDIATE ASSESSMENT  A268 = PAEDIATRICS - ENHANCED 18 MONTH WELL BABY VISIT  G538 = D&T IMMUNIZATION-WITH VISIT, EACH INJECT.  G539 = INJECTION OF UNSPECIFIED AGENT - SOLE REASON (FIRST INJECTION)  G590 = INFLUENZA AGENT +VISIT  G591 = INJECTION OF INFLUENZA AGENT - SOLE REASON  G840 = DTAPÛIPV-DIPHTHERIA, TETANUS, ACELLULAR PERTUSSIS, INACTIVATED POLIO VIRUS Û PAEDIATRIC  G841 = DTAPÛIPVÛHIBÙDIPHTHERIA, TETANUS, ACELLULAR PERTUSSIS, INACTIVATED POLIO VIRUS, HAEMOPHILUS INFLUENZA B  G842 = HBÙHEPATITIS B  G843 = HPVÙHUMAN PAPILLOMAVIRUS TYPE 6, 11, 16, 18  G844 = MENÛCÛCÙMENINGOCOCCAL C CONJUGATE  G845 = MMRÙMEASLES, MUMPS, RUBELLA  G846 = PNEU - PNEUMOCOCCAL CONJUGATE  G847 = TDAPÙTETANUS, DIPHTHERIA, ACELLULAR PERTUSSISÛADULT  G848 = VARÙVARICELLA  K017 = Periodic health visit – child  K130 = Periodic health visit – adolescent  K267 = ANNUAL HEALTH EXAM-CHILD-AFT. 2ND BIRTHDAY PAED.  K269 = ANNUAL HEALTH EXAM-PAEDIATRICS-ADOLESCENT-OFFICE  G592 = Administration of intranasal influenza vaccine  Q004 = CHILD IMMUNIZATION PREVENTIVE CARE SERVICE ENHANCEMENT  Q013 = NEW PATIENT FEE  Q015 = NEWBORN CARE EPISODIC FEE PCN  Q023 = UNATTACHED PATIENT FEE  Q033 = NEW GRAD/NEW PATIENT FEE  Q130 = INFLUENZA TRACKING CODE  Q132 = CHILDHOOD IMMUNIZATION TRACKING CODE  Q200 = PER PATIENT ROSTERING FEE - $5.00  Q590 = BASIC FLU SHOT FEE-PER-VISIT PREMIUM FHN/FHO  Q603 = INTERMED ASSESS- N.P.  Q613 = WELL BABY CARE - N.P. |
| Unscheduled primary care or minor visit billings | A001 = MINOR ASSESS.-F.P./G.P.  A003 = GEN. ASSESS. -F.P./G.P.  A004 = GEN.RE-ASSESS-F.P./G.P.  A005 = CONSULTATION -F.P./G.P.  A006 = RE-CONSULTATION-F.P./G.P.  A008 = MINI ASSESSMENT-F.P./G.P.  A261 = MINOR ASSESS.-PAED.  A901 = GENERAL/FAMILY PRACTICE-HOUSECALL ASSESSMENT  A903 = GEN/FAM PRACT-PRE-DENTAL/OPER.ASSESS LIMIT 2 PER YEAR/PT  A905 = GENERAL/FAMILY PRACTICE-LIMITED CONSULTATION  B960 = TRAVEL PREMIUM - SPECIAL VISIT TO PATIENT'S HOME - WEEKDAYS DAYTIME  B962 = TRAVEL PREMIUM - SPECIAL VISIT TO PATIENT'S HOME - EVENINGS M-F NON-ELECTIVE  B963 = TRAVEL PREMIUM - SPECIAL VISIT TO PATIENT'S HOME - SAT., SUN, HOLIDAYS NON-ELECTIVE  B964 = TRAVEL PREMIUM - SPECIAL VISIT TO PATIENT'S HOME - NIGHTS NON-ELECTIVE  B990 = SPEC VIS TO PT'S HOME, WK/DAYTIME  B993 = SPEC VIS TO PT'S HOME/NON-ELECT., SAT-SUN-HOLS  B994 = SPEC VIS TO PT'S HOME/NON-ELECT., EVE  B996 = SPECIAL VISIT-HOME-NIGHTS(12MN-7AM) 1ST PT.  E080 = 1ST VISIT PREM BY GP AFTER PAT IS DISCHARGED FROM HOSP  Q012 = AFTER HOURS PREMIUM  Q016 = AFTER HOURS PREMIUM – CCM  Q630 = REFERRAL TO SPECIALIST - N.P. |
| Other non-primary care visit billings | A013 = SPECIFIC ASSESS.-ANAES.  A015 = CONSULT.-ANAES.  A020 = COMPLEX DERMATOLOGICAL ASSESSMENT  A023 = SPECIFIC ASSESS.-DERM.  A024 = PARTIAL-ASSESS. -DERM.  A025 = CONSULT.-DERM.  A033 = SPECIFIC ASSESS.-GEN. SURG.  A034 = PARTIAL-ASSESS. -GEN. SURG.  A035 = CONSULT.-GEN. SURG.  A036 = RE-CONSULT.-GEN. SURG.  A043 = SPECIFIC ASSESS.-NEURO-SURG.  A045 = CONSULT.-NEURO-SURG.  A046 = RE-CONSULT.-NEURO-SURG  A063 = SPECIFIC ASSESS.-ORTHO-SURG.  A064 = PARTIAL-ASSESS. -ORTHO. SURG.  A065 = CONSULT.-ORTHO-SURG.  A066 = RE-CONSULT.-ORTHO-SURG.  A083 = SPECIFIC ASSESS.-PLASTIC SURG.  A084 = PARTIAL-ASSESS. -PLASTIC SURG.  A085 = CONSULT.-PLASTIC SURG.  A086 = RE-CONSULT.-PLASTIC SURG.  A180 = NEUROLOGY - SPECIAL NEUROLOGY CONSULTATION  A181 = COMPLEX MEDICAL SPECIFIC RE-ASSESSMENT  A183 = MEDICAL SPECIFIC ASSESSMENT  A184 = MEDICAL SPECIFIC RE-ASSESSMENT  A185 = CONSULT.-NEUROL.  A186 = RE-CONSULT.-NEUROL.  A188 = PARTIAL-ASSESS. -NEUROL.  A197 = CONSULT-INTERVIEW WITH PARENTS-PSYCHIATRY  A198 = CONSULT-INTERVIEW WITH CHILD-PSYCHIATRY  A203 = SPECIFIC ASSESS.-OBS.& GYN.  A205 = CONSULT.-OBS.& GYN.  A220 = GENETICS - SPECIAL GENETIC CONSULTATION  A223 = GENETICS - EXTENDED SPECIAL GENETIC CONSULTATION  A225 = CONSULTATION  A230 = ORTHOPTIC ASSESSMENT  A231 = NEURO-OPHTHALMOLOGY CONSULTATION  A233 = SPECIFIC ASSESS.-OPHTH.  A234 = PARTIAL ASSESS. -OPHTH.  A235 = CONSULT.-OPHTH.  A237 = PERIODIC OCULO-VISUAL ASSESS 19 YRS & UNDER  A243 = SPECIFIC ASSESS.-OTO.  A244 = PARTIAL ASSESS. -OTOL.  A245 = CONSULT.-OTO.  A246 = RE-CONSULT.-OTO.  A253 = OPHTHALMOLOGY - OPTOMETRIST - REQUESTED ASSESSMENT (ORA)  A263 = MEDICAL SPECIFIC ASSESSMENT-PAED  A264 = MEDICAL SPECIFIC RE-ASSESSMENT-PAED  A265 = CONSULT.-PAED.  A266 = RE-CONSULT.-PAED.  A284 = LABORATORY MEDICINE PARTIAL ASSESSMENT  A331 = MINOR ASSESSMENT DIAG RAD  A353 = SPECIFIC ASSESS.-UROL.  A354 = PARTIAL ASSESS. -UROL.  A355 = CONSULT.-UROL.  A356 = RE-CONSULT.-UROL.  A460 = COMPREHENSIVE INFECTIOUS DISEASES CONSULTATION  A461 = INFECTIOUS DISEASE - COMPLEX MEDICAL SPECIFIC RE-ASSESSMENT  A463 = INFECTIOUS DISEASE - MEDICAL SPECIFIC ASSESSMENT  A464 = INFECTIOUS DISEASE - MEDICAL SPECIFIC RE-ASSESSMENT  A465 = INFECTIOUS DISEASE - CONSULTATION  A466 = INFECTIOUS DISEASE - REPEAT CONSULTATION  A471 = COMPLEX MEDICAL SPECIFIC RE-ASSESSMENT  A473 = MEDICAL SPECIFIC ASSESSMENT  A474 = MEDICAL SPECIFIC RE-ASSESSMENT  A481 = COMPLEX MEDICAL SPECIFIC RE-ASSESSMENT  A483 = MEDICAL SPECIFIC ASSESSMENT  A565 = LIMITED CONSULT.-PAED.  A585 = DIAGNOSTIC CONSULT.-PATHOLOGY  A603 = MEDICAL SPECIFIC ASSESSMENT  A604 = MEDICAL SPECIFIC RE-ASSESSMENT  A605 = CONSULT.-CARDIOLOGY  A614 = MEDICAL SPECIFIC RE-ASSESSMENT  A623 = MEDICAL SPECIFIC ASSESSMENT  A628 = PARTIAL ASSESS. -CLIN. IMMUNOL.  A661 = COMPLEX MEDICAL SPECIFIC RE-ASSESSMENT  A765 = CONSULTATION, PATIENT 16 YEARS OF AGE AND UNDER  A813 = MIDWIFE REQUESTED ASSESSMENT  A815 = MIDWIFE REQUESTED SPECIAL ASSESSMENT  A888 = PARTIAL ASSESSMENT EM.DEPT EQUIVALENT  A900 = COMPLEX HOUSE CALL ASSESSMENT  A935 = PREAMBLE-SPECIAL SURGICAL CONSULT.  A960 = TRAVEL PREMIUM - SPECIAL VISIT TO PHYSICIAN OFFICE - WEEKDAYS DAYTIME(07:00 - 17:00)  A962 = TRAVEL PREMIUM - SPECIAL VISIT TO PHYSICIAN OFFICE- EVENINGS(17:00 - 24:00) M-F  A963 = TRAVEL PREMIUM - SPECIAL VISIT TO PHYSICIAN OFFICE- SAT., SUN AND HOLIDAYS(07:00 - 24:00)  A990 = SPEC VIS PHYS OFFICE - WK/DAYTIME  A993 = A993 - DESCRIPTION UNKNOWN  A998 = SPECIAL VISIT PREMIUM PHYSICIAN OFFICE - SAT., SUN. AND HOLIDAYS (07:00 - 24:00)  B997 = SPEC VIS PALLIATIVE CARE HOME, DAYS, EVE  E078 = MEDSPECASSESS&RE-ASSESS,COMPLEXMEDSPEC RE-ASSESS&PART ASSES  E423 = MANUAL CYCLOPLEGIC REFRACTION, TO A233, A234  G556 = ICU/NICU ASSESSMENT FEE WHEN INITIAL VISIT RENDERED DURING NIGHT TIME  G557 = D/T PROC.COMPREHEN.INTENS.CRIT.VENT.SUP.PHYS.IN CHGE-1STDAY  G558 = D/T PROC.COMP.INTENS.CRIT.VENT.PHYS.IN CHGE 2NDTO10THDAY  G559 = COMPR.INTENS.CARE CRIT.&VENT.SUP.PHYS.IN CHGE.11TH DAY ONWAR  G611 = D./T. PROC. NEONATAL INTENSIVE CARE-LEVEL B DAY 2 ONWARD  G621 = D./T. PROC. NEONATAL INTENSIVE CARE-LEVEL C DAY 2 ONWARD  K399 = ALLERGY-CLINICAL INTERP./IMMUNOL/REPORT IN WRITING  K704 = PAEDIATRIC OUT-PATIENT CASE CONFERENCE  K730 = TELEPHONE CONSULTATION - PHYSICIAN TO PHYSICIAN - REFERRING PHYSICIAN  K731 = TELEPHONE CONSULTATION - PHYSICIAN TO PHYSICIAN - CONSULTANT PHYSICIAN  K732 = TELEPHONE CONSULTATION Û CRITICALL - REFERRING PHYSICIAN  K734 = EMERG DEPART - PHYSICIAN TO PHYSICIAN TELEPHONE CONSULTATION – REF PHYSICIAN  K735 = EMERG DEPART - PHYSICIAN TO PHYSICIAN TELEPHONE CONSULTATION – CONSULT PHYSICIAN  K738 = PHYSICIAN TO PHYSICIAN E-CONSULTATION - REFERRING PHYSICIAN  K739 = PHYSICIAN TO PHYSICIAN E-CONSULTATION - CONSULTANT PHYSICIAN  U025 = DERMATOLOGY - INITIAL E-ASSESSMENT  U960 = TRAVEL PREMIUM - SPECIAL VISIT TO HOSPITAL OUT-PT DEPT - WEEKDAYS DAY  U961 = TRAVEL PREMIUM - SPECIAL VISIT TO HOSPITAL OUT-PT DEPT - WEEKDAYS DAY SAC OFFICE HRS  U962 = TRAVEL PREMIUM - SPECIAL VISIT TO HOSPITAL OUT-PATIENT DEPARTMENT - EVENINGS M-F  U963 = TRAVEL PREMIUM - SPECIAL VISIT TO HOSPITAL OUT-PATIENT DEPARTMENT – SAT SUN HOL  U990 = SPEC VIS - HOSP OUT-PT. - WK/DAYTIME  U991 = SPEC VIS - HOSP OUT-PT. -SK/DAYTIME, ADDIT'L PT.  U992 = SPEC VIS- HOSP OUT-PT.-SAC.OFF.HRS. WK/DAYTIME  U993 = SPEC VIS- HOSP OUT-PT., MON-FRI., EVE  U994 = OPD SPECIAL VISIT PREMIUM CODES  U995 = SPEC VIS- HOSP OUT-PT., MON-FRI., EVE, ADDIT'L PT.  U997 = SPEC VIS - HOSP OUT-PT., NIGHTS, ADDIT'L PT.  U998 = SPECIAL VISIT PREMIUM HOSPITAL OUT-PT DEPT – SAT SUN HOLIDAYS - FIRST PATIENT SEEN  U999 = SPECIAL VISIT PREMIUM HOSPITAL OUT-PT DEPT - ADDITIONAL PERSON(S) SEEN |
